# Zooplankton Metabolism Shapes Molecular Composition of Dissolved Organic Matter in Coastal Waters

**DOI:** 10.1021/acs.est.6c05592

**Published:** 2026-05-06

**Authors:** Muhammad Firman Nuruddin, Yiu Cho Joe Poon, Xuejia He, Zekun Zhang, Xiaoming Tang, Sangwook Scott Lee, Shuwen Zhang, Ding He, Longjun Wu

**Affiliations:** † Department of Ocean Science, 58207 The Hong Kong University of Science and Technology, Hong Kong, SAR 999077,China; ‡ Center for Ocean Research in Hong Kong and Macau, Hong Kong, SAR 999077, China; § College of Life Science and Technology, Key Laboratory of Eutrophication and Red Tide Prevention of Guangdong Higher Education Institutes, 506476 Jinan University, Guangzhou 510632, Guangdong, China; ∥ Guangdong Provincial Key Laboratory of Biotechnology for Plant Development, Guangzhou Key Laboratory of Subtropical Biodiversity and Biomonitoring, 12451 South China Normal University, West 55 of Zhongshan Avenue, Guangzhou 510631, Guangdong, China

**Keywords:** Zooplankton, Dissolved Organic Matter, Meta-transcriptomics, FT-ICR MS, Gene Expression, Metabolism, Biogeochemical Cycle

## Abstract

Dissolved organic matter (DOM), the largest reservoir of organic material in the ocean, plays a crucial role in the global nutrient cycle and the microbial loop. While existing studies have documented significant DOM release by marine organisms, how organismal metabolic processes under different nutrient levels influence the intrinsic characteristics of their derived DOM remains largely unknown. We conducted zooplankton DOM release experiments in estuarine-coastal water systems, followed by molecular characterization of the DOM using Fourier transform ion cyclotron resonance mass spectrometry and metabolic profiling using community-level gene expression analysis (metatranscriptomics). Our results reveal substantial differences in the molecular composition and characteristics of zooplankton-derived DOM from eutrophic compared to mesotrophic communities. Moreover, zooplankton in mesotrophic environments exhibited a higher expression of metabolic genes. We found strong correlations between the zooplankton-derived DOM chemical composition and zooplankton gene expression associated with metabolic processes including carbohydrate metabolism, amino acid, energy production, lipid, terpenoid-polyketide and glycan biosynthesis. Furthermore, the modified aromaticity indexes of the zooplankton-derived DOM are also highly associated with metabolism-related gene functions, suggesting that metabolic responses may drive the derived DOM aromaticity characteristics. This study enhances our understanding of how organismal metabolic activities shape the molecular properties of released DOM, underscoring their critical roles in biogeochemical cycling.

## Graphical abstract

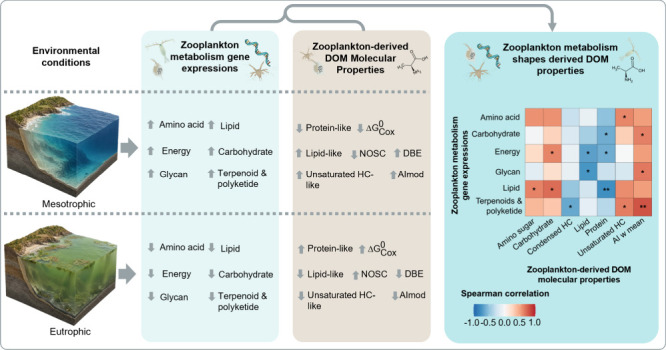

## Introduction

Dissolved organic matter (DOM) constitutes Earth’s largest reservoir of organic carbon, driving marine biogeochemical cycles, regulating nutrient bioavailability, and sustaining microbial food webs via the microbial loop.^1,2^ Beyond its substantial carbon content, DOM is a reservoir of nitrogen, phosphorus, and trace metals, underscoring its critical role in oceanic elemental cycling.^3^ A diverse array of DOM is actively produced by marine organisms through processes such as photosynthesis, cell lysis, viral shunt, grazing, and the dissolution of zooplankton excreta, thereby supporting heterotrophic metabolism.^4–6^ While current studies focus on the characterization,^7^ transformation,^8^ and degradation^9,10^ of DOM in the environment, the biological mechanisms driving DOM production remain poorly understood. A critical question is how organisms’ metabolic responses to environmental heterogeneity influence the molecular properties of the DOM they release. Resolving this question is essential for advancing our understanding of DOM dynamics and its role in the biogeochemical cycling.

Zooplankton are a diverse group of heterotrophic plankton, including copepods, cladocerans, and gelatinous animals. They occupy a central trophic position in aquatic ecosystems, linking primary producers to microbial loops.^1,11–14^ Up to 50% of daily primary production in the ocean is consumed by zooplankton, and 10–30%^12^ of this grazed material is released as DOM through processes such as excretion, sloppy feeding, and byproducts of digestion.^13–17^ In addition to releasing DOM, zooplankton are also capable of directly assimilating dissolved organic substrates.^18,19^ These integrated processes underscore the important role of zooplankton in mediating both the production and transformation of DOM within aquatic food webs. Compared to the primary producer in the ocean, zooplankton release a biochemically distinct suite of DOM. Whereas phytoplankton DOM is typically enriched in simple sugars, nitrogenous compounds, and organic acids,^20–22^ zooplankton-derived DOM includes metabolic products such as dissolved free amino acids, fatty acids, and proteins. These compounds provide high-quality substrates for prokaryotic communities and support diverse microbial populations.^15,16,23^ Collectively, these compositional differences indicate that zooplankton-derived DOM disproportionately contributes to the bioavailable DOM pool.

In addition to the unique composition of zooplankton-derived DOM relative to other autochthonous DOM, zooplankton possess substantially larger body sizes compared with phytoplankton and microbes. This trait enables more robust metabolic analyses, for example, extracting sufficient RNA from a single individual, and facilitates straightforward physical separation from other planktonic components. These characteristics facilitate the investigation of zooplankton metabolism and the isolation of zooplankton-derived DOM. Therefore, beyond their important ecological roles, zooplankton represent a promising experimental system for elucidating how organismal metabolic processes modulate the molecular characteristics of their derived DOM.

Advances in ultrahigh-resolution mass spectrometry and community-level omics now enable unprecedented exploration of DOM and organism metabolic process linkage. Techniques such as Fourier Transform Ion Cyclotron Resonance Mass Spectrometry (FT-ICR MS) allow for the detailed characterization of DOM molecular formulas.^7,24^ Concurrently, metatranscriptomics, which involves the study of gene expression at the community level, enables examination of metabolic processes in the whole community.^25,26^ In this study, we integrate these methodologies to elucidate the connection between organisms’ metabolic responses and their derived DOM characteristics. We conducted spatially resolved zooplankton sampling and DOM release incubation along the Pearl River—China’s second largest river—Estuary and adjacent coastal regions. Our approach reveals distinct molecular characteristics of zooplankton-derived DOM in mesotrophic and eutrophic conditions. Additionally, we discovered differential expression of metabolic genes across nutrient gradients. Finally, we observe strong linkages between DOM chemical signatures (including molecular composition and aromaticity characters) and zooplankton metabolic gene functions (including metabolisms of amino acid, carbohydrate, lipid, energy production, terpenoid-polyketide, and glycan biosynthesis). These findings not only reveal the distinct characteristics of zooplankton-derived DOM in estuarine-coastal environments but also demonstrate how metabolic responses strongly influence DOM characteristics. Together, they provide new mechanistic insight into how organismal metabolism shapes DOM dynamics in aquatic systems.

## Materials and Methods

### Zooplankton Field Sampling

We collected zooplankton samples during summer 2023 (August) and 2024 (July) cruises in Pearl River Estuary-Hong Kong coastal waters from the surface (2 m depth, neustonic layer) layers using a custom-made plankton pump equipped with a 150 μm mesh sieve. The zooplankton samples were collected by deploying the plankton pump for 15 min with a pumping rate of 105 m^3^/h. The sampling sites were selected based on the long-term monitoring stations of the Environmental Protection Department of Hong Kong Monitoring Program for the Summer 2023 and Earth HK project Cruise stations for Summer 2024. We categorized the sampling sites into two distinct physicochemical zones (Figure 1a) — mesotrophic and eutrophic — based on the TRIX trophic index (Supporting Information Text S1 for the details of the equation), a well-established metric for evaluating aquatic trophic status.^27,28^

**Figure 1:**
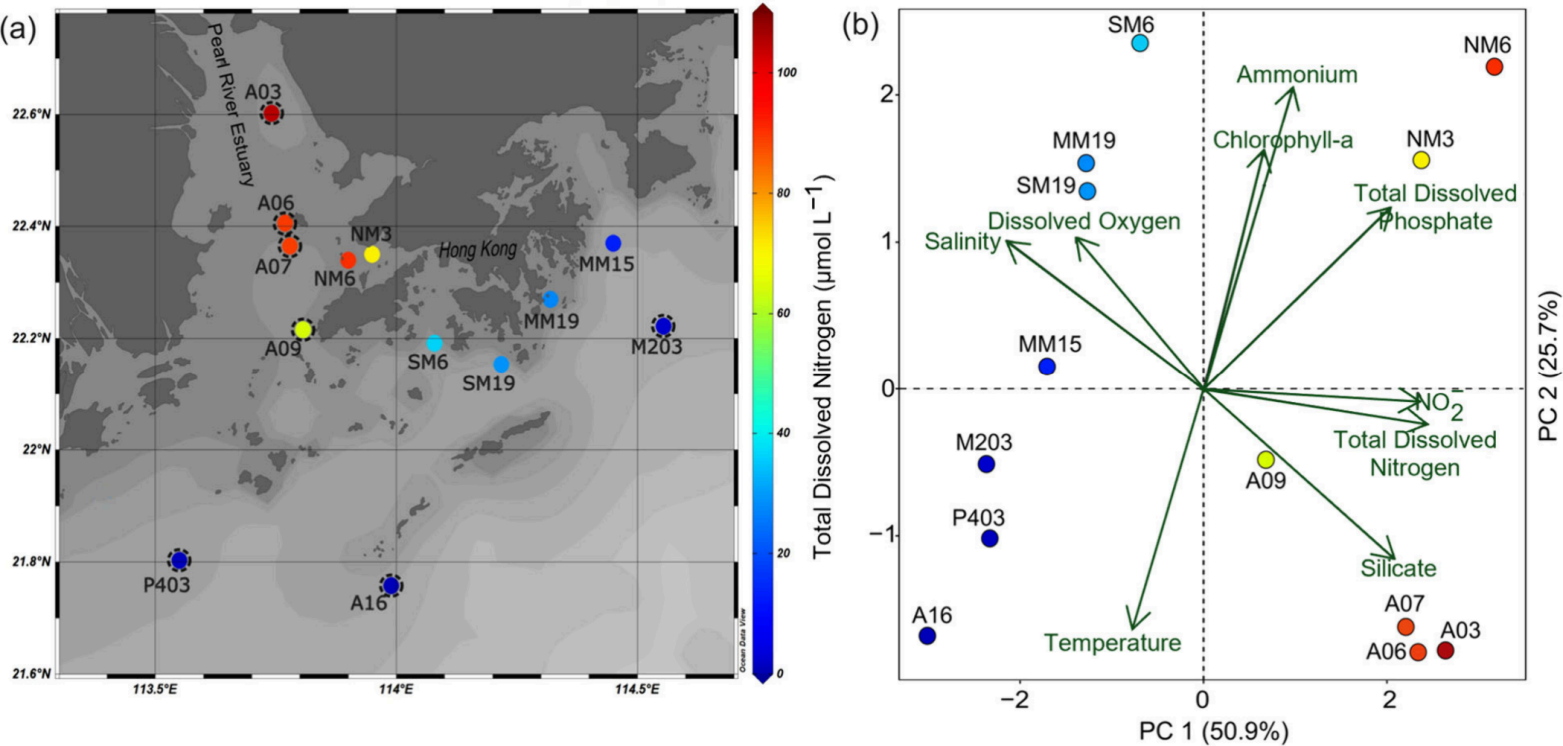
Sampling sites and their environmental conditions. (a) Location of the zooplankton sampling sites along the Pearl River Estuary and adjacent coastal waters. (b) Principal component analysis (PCA) biplot of environmental parameters. The stations for zooplankton-derived DOM release incubation were indicated by dashed circles. The colors of dots denote Total Dissolved Nitrogen (μmol L^–1^), while the arrow length represents the contributions of each environmental parameter toward the variance of the principal component. The station naming codes are as follows: A, M, and P refer to stations along transects A, M, and P during the cruise, respectively; NM, MM, and SM denote stations in the Northern, Mirs Bay, and Southern Hong Kong monitoring regions, respectively.

### Zooplankton-Derived Dissolved Organic Matter Release Experiment

We conducted incubation experiments to evaluate the release of DOM by zooplankton during the Summer Cruise 2024. Initially, the collected zooplankton samples were rinsed with 0.22 μm-filtered ambient seawater to minimize the bacteria attached to the zooplankton body. Subsequently, we incubated the zooplankton in a 2-L clean water bottle containing 1 L of 0.22 μm-filtered ambient seawater. For comparison, a control treatment consisting of 0.22 μm-filtered ambient seawater without zooplankton was also incubated. The incubation lasted for 8 hours in the dark at in situ temperature (Supporting Information Figure S1). We performed a visual inspection of the zooplankton before and after the incubation to ensure they remained in a healthy condition. Following the incubation period, we separated the zooplankton from the released DOM using a 0.22 μm polycarbonate Isopore membrane filter. The collected DOM was then preserved at −20 °C. This incubation experiment was conducted in triplicate to ensure the reliability of the results. While we acknowledge that bacteria could still influence DOM composition during incubation, we minimized this effect by thoroughly rinsing zooplankton to remove as many attached bacteria as possible. Some microbes inevitably remain, particularly those residing within the zooplankton gut. However, these communities are naturally associated with zooplankton in situ. We consider the DOM produced during incubation to be zooplankton-derived.^17^ Additionally, the relatively short incubation period may further minimize bacterial transformation of zooplankton-derived DOM.^23,29^

To identify the unique DOM molecular formulas derived from zooplankton, we compared the zooplankton incubation group with the corresponding control group. Specifically, zooplankton-derived DOM was determined by subtracting the molecular formulas identified in the zooplankton treatment from the molecular formulas detected in the control incubation (filtered in situ seawater without zooplankton) after incubation.^23^ This subtraction aims to remove the background DOM signal and isolate the contribution of zooplankton.

### DOM Extraction and Characterization by FT-ICR-MS

We used solid-phase extraction (SPE) to extract DOM from the 0.22 μm-filtered incubation water samples following well-established protocols.^30^ In summary, 500 mL of samples was filtered through GTTP filters (Millipore, 0.22 μm pore size, which were prerinsed three times with Milli-Q water). The samples were then acidified to a pH of 2 using analytical-grade HCl and passed through a 1-g SPE cartridge (which had been prerinsed twice with methanol, Milli-Q water, and Milli-Q water at pH 2) (200 mg, Bond Elut, PPL, Agilent). The cartridges were desalted using 0.01 M HCl and dried with pure nitrogen gas. Finally, the SPE-DOM samples were eluted from the cartridge with 2 mL of 99.9% HPLC-grade methanol.

To characterize the molecular formulas of the SPE-DOM, methanol PPL extracts were analyzed on a 15 T Solarix FT-ICR MS (Bruker Daltonik GmbH, Bremen, Germany) with electrospray ionization source in negative mode. FT-ICR MS acquisition and data processing followed established community workflows for DOM molecular characterization, including instrument blank correction, signal-to-noise filtering, mass calibration, spectral alignment, and molecular formula assignment based on validated protocols.^7,31–36^ Briefly, 200 scans were used in the mass range *m*/*z* 100 to 1000 Da with the calibration of a series of established formula mass peaks (Bruker Compass Data Analysis 5.0 software package).^34^ Peaks were selected when the signal/noise ratio (S/N) was greater than 6. Formulas were assigned according to the formula rules,^37^ allowing for elemental compositions of C_1–100_H_1–200_O_1–50_N_0–3_P_0–1_S_0–2_ with an error range of ±1 ppm.

The annotated zooplankton-derived DOM molecules were assigned to biochemical compound categories based on the stoichiometry of C, H, and O for the following H/C and O/C ranges: lipids (0 < O/C ≤ 0.3, 1.5 ≤ H/C ≤ 2.5), unsaturated hydrocarbons (0 ≤ O/C ≤ 0.125, 0.8 ≤ H/C < 2.5), proteins (0.3 < O/C ≤ 0.55,1.5 ≤ H/C ≤ 2.3), amino sugar (0.55 < O/C ≤ 0.7, 1.5 ≤ H/C ≤ 2.2), carbohydrates (0.7 < O/C ≤ 1.5, 1.5 ≤ H/C ≤ 2.5), lignin (0.125 < O/C ≤ 0.65, 0.8 ≤ H/C < 1.5), tannin (0.65 < O/C ≤ 1.1, 0.8 ≤ H/C < 1.5), and condensed hydrocarbons (0 ≤ O/C ≤ 0.95, 0.2 ≤ H/C < 0.8). Unnamed compounds (others) are calculated as the proportion of identified formulas that do not fit within the above-defined H/C and O/C ranges.^38^ Relative peak intensities were calculated based on the sum-normalized intensities of all assigned peaks in each sample.^55^ Moreover, while FT-ICR-MS provides a highly detailed, untargeted representation of SPE-DOM fraction, it should be noted that some larger DOM biopolymers may fall outside the analytical window of this approach. Therefore, in this study, we focus on the fraction of DOM that is captured by this routine analytical approach, representing >60% of marine DOM.^30^

### Zooplankton Preservation and Environmental Parameters

We performed filtration using a 10 μm nylon membrane to separate the collected zooplankton from the particulate matter. The filters containing zooplankton were preserved in Invitrogen RNAlater Stabilization Solution, a solution to prevent RNA from degradation, and stored at −20 °C until RNA extraction in the laboratory. Concurrently, water quality parameters including temperature (°C), salinity (PSU), and dissolved oxygen (%) were measured at each sampling station using a multiparameter CTD-DO prior to zooplankton sampling. Additionally, we collected water samples for nutrient and chlorophyll-a analyses. Nutrient samples were filtered through a 0.45 μm acetate fiber membrane, while chlorophyll-a samples were obtained by filtering through a GF/F membrane. Nutrient concentrations including nitrate (NO_3_^–^), nitrite (NO_2_^–^), ammonium (NH_4_^+^), soluble reactive phosphate (PO_4_^3–^), and silicate (SiO_3_^2–^) (all expressed in μmol L^–1^) were measured spectrophotometrically on a Seal 5-channel AA500 AutoAnalyzer following the Environmental Protection Agency standard protocols.^39,40^ Chlorophyll-a was measured by following the method proposed by SCOR-UNESCO.^41^ Meanwhile, for the 2023 samples, we utilized complementary environmental data collected at the same sampling stations during the same month by the Environmental Protection Department (EPD) of Hong Kong. This data is publicly available through the EPD Data Repository at the Environmental Protection Interactive Centre, specifically under the Marine Water Quality Monitoring Data. In addition to physical and chemical environmental parameters, we also collected phytoplankton samples in each sampling station with detailed methods provided in Supporting Information Text S2.

### RNA Extraction, Library Preparation, and Sequencing

The collected zooplankton samples preserved in RNAlater were used for zooplankton total RNA extraction by using Vazyme FastPure Cell/Tissue Total RNA Isolation Kit V2 following the manufacturer’s protocol. For the purpose of RNA quantity and quality checking, Qubit fluorometer, Biodrop, and Gel Electrophoresis were used prior to library creation and sequencing. In the RNA sequencing step, each sample was processed by isolating mRNA from one microgram of total RNA through poly-T oligo-attached magnetic bead selection. Subsequently, the mRNAs were reverse transcribed into cDNAs to construct sequencing libraries with average insert sizes ranging from 250 to 300 bp, utilizing NovaSeq X Series 25BRNA Sample Prep Kits (300 cycle) in accordance with the manufacturer’s guidelines. To ensure robustness and account for biological variability, two to three biological replicates were included for each sample, resulting in a total of 34 cDNA libraries. The cDNA libraries were subjected to pair-end sequencing, producing 150 bp reads using the NovaSeq Xplus sequencing platform (Illumina) following standard protocols.

### Metatranscriptome De Novo Assembly, Transcript Abundance Estimation, and Functional Annotation

The raw RNA sequence data quality was evaluated using FastQC Version 0.11.5,^42^ followed by quality trimming of the raw reads for poor quality bases and sequencing adapters using Trimmomatic.^43^ The resulting paired-end clean sequence from all 34 cDNA libraries was simultaneously utilized to construct the metaassembly using Trinity software v2.15.2 ^44^ with default settings, yielding 12,975,434 contigs. The assembled contigs are then used for open reading frame prediction by using transdecoder^45^ against pfam database.^46^ Subsequently, we performed transcript abundance quantification using the Salmon-based approach.^47^ In brief, the reads are first aligned to the assembled contigs using Bowtie2.^48^ The output BAM file is then used as the input for Salmon^47^ tool. Normalization of transcript per million (TPM) was performed based on library sizes using the “calcNormFactors (method = “TMM”)” function to ensure accurate quantification of gene expression levels across the samples.^49^

To obtain functional annotations for the assembled contigs, we utilized the Eggnog-mapper pipeline.^50^ This process required protein sequence as one of the inputs for the functional annotations; consequently, we used Transdecoder^45^ software packaged with Trinity^44^ to predict the protein translations from the contigs. Subsequently, the unigenes obtained were queried against several public databases, including Swiss-Prot, Pfam-A, and Eggnog,^51^ with an e-value threshold of 1E^–5^. Next, to obtain the taxonomy origins of our metatranscriptome, we annotated the taxonomy compositions of assembled contigs toward the NCBI nonredundant protein database^52^ using the MMseqs2 ^53^ last common ancestor algorithm. On the basis of these assignments, transcripts annotated as originating from bacteria, prokaryotes, or phytoplankton were explicitly removed prior to downstream functional and network analyses. This filtering step was applied to ensure that subsequent analyses captured zooplankton-derived gene functions and minimized the potential contributions from associated microbial or algal taxa. Only genes retained after taxonomic filtering were used for all downstream analyses, including KEGG enrichment, WGCNA, and calculation of the transcriptome metabolic index (TMI). The full annotation table of retained metazoan-assigned genes used in this study can be accessed through 10.5281/zenodo.19343331.

### Putative DOM Biochemical Reactions Analysis

Putative biochemical reactions within dissolved organic matter (DOM) were inferred from ultrahigh-resolution mass spectrometry data using established methodologies.^54^ Pairwise mass differences among all retained peaks were then calculated and compared against a reference library of 100 common chemical transformations^55^ (provided in 10.5281/zenodo.17156986) under a mass error tolerance of ±0.5 ppm. Matching mass differences were interpreted as putative gains or losses of specific compounds via chemical reactions.^56^ Although elemental formula differences do not always guarantee that the corresponding molecular structures can undergo a given transformation, it remains a powerful method for identifying potential biochemical linkages and comparing transformation patterns across environmental conditions.^57–62^

### Statistical Analyses and Data Visualization

We employed the MetaboDirect pipeline^55^ to conduct data exploration and statistical analysis of the DOM data, including the generation of Van Krevelen diagrams, Venn diagrams, and visualizations of DOM relative abundance. The input data consisted of preprocessed DOM molecular formulas along with their corresponding monoisotopic peak intensities and *m*/*z* values. To evaluate molecular properties and potential decomposability, we computed several thermodynamic and molecular indices based on each peak’s elemental composition (equations provided in Supporting Information Table S2). These indices included the Nominal Oxidation State of Carbon (NOSC), which describes the average carbon oxidation state of the DOM molecules; Gibbs energies of the oxidation half reactions (ΔG_Cox_^0^), indicating the thermodynamic favorability of degradation;^63^ the Modified Aromaticity Index (AImod), reflecting aromaticity and carbon double-bond density;^64^ and the double bond equivalent (DBE), quantifying molecular unsaturation as a proxy for DOM aromatic structures.^65^

During data exploration, the MetaboDirect pipeline generated Van Krevelen diagrams^55^ to visualize molecular formula distributions, along with violin plots of the calculated molecular indices. Statistical differences between eutrophic and mesotrophic zooplankton-derived DOM were assessed using Tukey post hoc tests, while box plots summarized molecular and elemental compositions across different regions. Additionally, pairwise comparisons were performed to identify unique and shared molecules between mesotrophic and eutrophic groups, employing van Krevelen diagrams, Venn diagrams, and stacked bar plots for visualization.

To assess zooplankton metabolic responses to environmental heterogeneity, we performed several analyses of genes annotated to the KEGG database. First, we performed Spearman correlation analyses to examine the relationship between metabolic gene functions and environmental parameters. This initial analysis aimed to identify the general metabolic responses of zooplankton to varying environmental conditions. Subsequently, we sought to further investigate the coexpression of metabolic function genes and their relationships with environmental variables. We conducted a weighted gene coexpression network analysis (WGCNA) using the WGCNA package (version 1.72–1) in R.^66^ We used a trimmed mean of M-values (TMM) normalized expression matrix of KEGG-annotated genes as input to identify gene modules exhibiting strong coexpression patterns. The network construction employed an unsigned topological overlap matrix (TOM), with module detection based on TOM-derived dissimilarity measures (1-TOM). We optimized various parameters for biological network properties, including a soft threshold power (β) of 30 to meet scale-free topology criteria, a minimum module size of 70 genes, and a merge cut height of 0.4.^67^ Module eigengenes were computed and correlated with environmental parameters. To identify metabolic pathways associated with significant module eigengenes, we performed gene enrichment analysis using the clusterProfiler package^68^ in R. Resulting p-values were adjusted for multiple comparisons using the Benjamini-Hochberg false discovery rate (FDR) correction.^67,69^ Finally, we developed a Transcriptome Metabolic Index (TMI) to compare zooplankton metabolic processes between trophic states. TMI is a complementary, systems-level metric intended to summarize aggregate metabolic gene expression, rather than identify individual differentially expressed genes. Specifically, we calculated the eutrophic-to-mesotrophic ratios of total transcripts per million (TPM) values for each KEGG metabolic subcategory using formula [Disp-formula eq1], where i equals 1 through n represents the KEGG metabolism from all the annotated gene lists (gene 1 to n).

$$\mathrm{TMI}=\frac{\overset{n}{\underset{i=1}{\sum }}\mathrm{TPM}\;\mathrm{of}\;\mathrm{KEGG}\;\mathrm{metabolism}\;\mathrm{in}\;\mathrm{Eutrophic}}{\overset{n}{\underset{i=1}{\sum }}\mathrm{TPM}\;\mathrm{of}\;\mathrm{KEGG}\;\mathrm{metabolism}\;\mathrm{in}\;\mathrm{Mesotrophic}}$$ 1

To examine the link between phytoplankton composition, zooplankton metabolic gene profiles, and DOM molecular classes, we conducted a Mantel test analysis using the linkET R package version 0.0.3.^70^ Subsequently, Spearman rank correlation analysis between zooplankton metabolic genes profiles and DOM chemical characteristics (molecular compositions and intrinsic characters) was also performed using the R cor­() function.^71^ For this analysis, we focused on zooplankton metatranscriptome samples collected from sites where we conducted DOM incubation, allowing us to correlate zooplankton gene expression with DOM properties effectively. Next, we tested the mechanistic basis of the observed correlation by checking the association between DOM molecular transformation and the zooplankton gene expression profiles through another Spearman correlation approach.

## Results and Discussion

### Zooplankton-Derived DOM Composition and Characteristics

To characterize the chemical properties of DOM derived from zooplankton in heterogeneous coastal waters, we conducted onboard DOM release incubation experiments (see Materials and Methods for details). These incubation experiments generated zooplankton-derived DOM samples across sites spanning from the Pearl River Estuarine Delta to adjacent coastal waters (Figure 1a).

To identify the key environmental heterogeneity across the sampling stations, we performed a principal component analysis (PCA) on measured environmental parameters (Figure 1b). The first two principal components (PC1 and PC2) collectively explained 76.6% of the total variance across the sampling sites. PC1 was primarily driven by nutrient concentrations, including total dissolved nitrogen and nitrite (NO_2_^–^), followed by physical factors, more specifically, salinity. Meanwhile, PC2 reflected variations in the levels of ammonium, total dissolved phosphate, and temperature. Given the distinct nutrient gradients observed and their dominant contribution to the overall heterogeneity of environmental parameters in the sampling locations, we categorized sampling stations based on trophic state indices, revealing a clear separation into mesotrophic (blue dots) and eutrophic (yellow to red dots) regions (Figure 1a). Therefore, our following analysis focuses on observing the overall composition of zooplankton-derived DOM across the nutrient gradients from mesotrophic to eutrophic stations.

We retrieved the molecular formula of DOM from both the control and zooplankton incubation treatments by FT-ICR MS. Next, we operationally characterized zooplankton-derived DOM from our onboard incubation by subtracting the molecular formula identified in zooplankton treatments from that of their corresponding control. A total of 4,945 unique molecular formulas were identified with an average of 900 formulas per incubation. To provide an overview of the compositional differences between control and zooplankton-derived DOM, we performed a PCA on the identified DOM molecular formula (Supporting Information Figure S3). The PC1 contributed 57.16% and the PC2 contributed 14.5% of the total molecular formula variation, respectively. These patterns underscore the clear role of zooplankton in enriching the DOM pool with chemically distinct metabolites.^17^

Subsequently, we operationally classified the zooplankton-derived DOM into major biochemical categories such as lipids, proteins, amino sugars, carbohydrates, unsaturated hydrocarbons, lignin, tannins, and condensed hydrocarbon-like compounds, based on their H/C and O/C ratios threshold, as illustrated in the Van Krevelen diagram (Figure 2a). Nearly half (48.8%) of the DOM molecules were detected in both eutrophic and mesotrophic zooplankton communities, while 30.8% and 20.5% were unique to eutrophic and mesotrophic systems, respectively (Figure 2b). This overlap pattern suggests a core set of metabolic byproducts consistently released by zooplankton across nutrient regimes, with unique molecular signatures that may reflect the outcome of environment-specific physiological processes.^72^ Across all regions, lignin-like molecules dominated the molecular composition (55–62%), followed by protein-like (12–20%), condensed hydrocarbon-like (5–15%), and tannin-like (∼5%) compounds (Figure 2c), highlighting the chemically complex nature of zooplankton-derived DOM.

**Figure 2:**
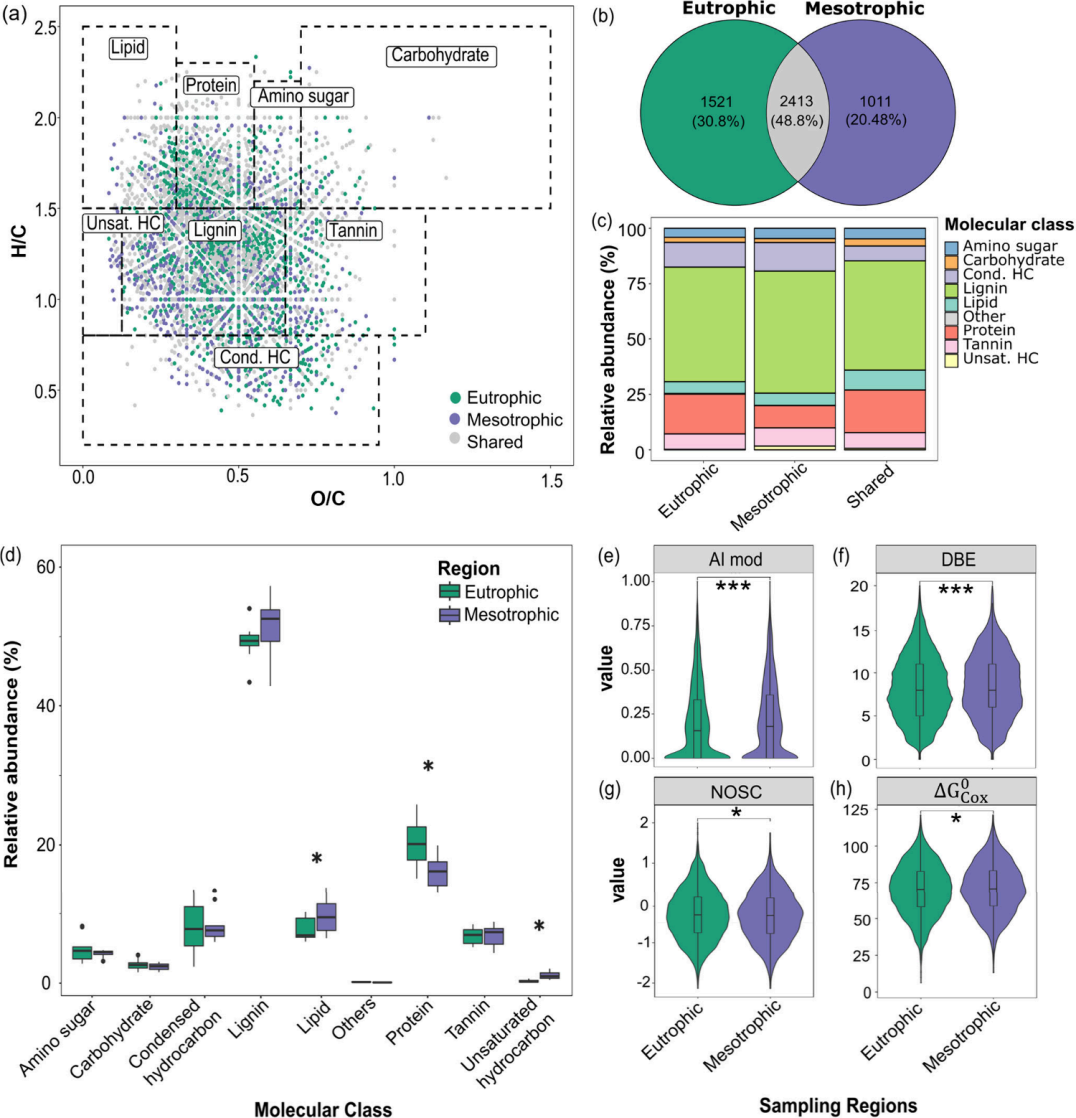
Distributions of zooplankton-derived DOM molecules. (a) Van Krevelen diagram of the DOM molecules with the colors showing the molecular formula that are shared and unique between eutrophic and mesotrophic regions. (b) Venn diagram of detected molecular formula numbers in each region (mesotrophic, eutrophic, and shared). (c) Relative abundance of DOM compounds like composition, which was uniquely detected in eutrophic and mesotrophic regions and shared in both regions, with colors representing different molecular compound classes. Comparison of zooplankton-derived DOM molecules properties between eutrophic and mesotrophic. (d) Relative abundance (%) of DOM molecular classes with the clear difference’s molecule classes marked by an asterisk. DOM chemical (e) Modified aromaticity indexes (AImod), (f) Double Bond Equivalent (DBE), (g) Nominal Oxidation State of Carbon (NOSC), and (h) Gibbs energies of the oxidation half reactions (Δ*G*_*Cox*_^0^). The colors represent the eutrophic and mesotrophic regions. Significant differences (* = p value <0.05, *** = p value <0.001) are indicated by the asterisks.

To resolve the uniqueness of zooplankton-derived DOM relative to other biological sources, we compared its composition with DOM released by three representative phytoplankton groups: *Synechococcus* sp. (cyanobacteria), *Prorocentrum donghaiense* (diatom), and *Skeletonema costatum* (dinoflagellate).^73,74^ Zooplankton-derived DOM contained higher proportions of amino sugar-, carbohydrate-, and tannin-like molecules than phytoplankton-derived DOM (Supporting Information Figure S4). In contrast, lignin-like molecules were more abundant in the phytoplankton DOM. This compositional difference indicates that zooplankton release a suite of metabolites that differ fundamentally from those of phytoplankton exudates. These compounds likely originate from zooplankton digestive processes, mucus production, and tissue turnover.^4,12^ Our results align with previous studies reporting that zooplankton-derived DOM contains complex molecules, such as amino acids, fatty acids, and protein-like compounds.^15,16,23^ The substantial compositional distinctions suggest that zooplankton-derived DOM plays unique roles in biogeochemical cycling, particularly by contributing more nitrogen-rich and structurally diverse substrates to the DOM pool.^17,23,57^ The higher abundance of amino sugar- and carbohydrate-like molecules, in particular, implies an enhanced potential for microbial utilization.^15,23^ Together, these features highlight zooplankton as important autochthonous DOM contributors to DOM chemical diversity in coastal ecosystems.

Although the contribution of zooplankton to shaping DOM composition has been investigated,^17,23,75^ the pattern of zooplankton-derived DOM in different trophic levels remains largely unknown. Thus, we compared the molecular composition of zooplankton-derived DOM between mesotrophic and eutrophic regions. Mesotrophic zooplankton-derived DOM exhibited significantly higher proportions of lipid-like and unsaturated hydrocarbon-like compounds, whereas eutrophic DOM was enriched in protein-like molecules (Figure 2d).

Since DOM’s intrinsic molecular characteristics such as degree of unsaturation, redox properties, as well as aromaticity signatures influence DOM fate, stability, and lead to profound impacts on biogeochemical cycling,^3^ we compared these features between zooplankton-derived DOM from mesotrophic and eutrophic environments. Mesotrophic zooplankton-derived DOM displayed clearly higher modified aromaticity indices and double bond equivalent values (Figure 2e and f), indicative of structurally complex, aromatic, and unsaturated DOM molecules. Such compounds are typically more biologically recalcitrant, aligning with relatively lower Gibbs energies of the oxidation half reactions values (Figure 2h), which suggest reduced bioavailability and slower microbial degradation rates.^65,76^ In contrast, eutrophic zooplankton-derived DOM showed a higher nominal oxidation state of carbon (NOSC; Figure 2g), indicating enhanced oxidative potential and labile substrates. These findings offer important insights into the variations of zooplankton-derived DOM molecular composition and properties under different trophic conditions, which would affect their fate and cycle in the environment.^15,17,23,57^

### Zooplankton Metabolic Profiles Are Highly Associated with Environmental Factors

We characterized the differences in zooplankton-derived DOM between eutrophic and mesotrophic conditions. It is also essential to understand how zooplankton respond internally to these varying conditions, as their metabolic responses can be an important factor driving differences in zooplankton-derived DOM properties.^4^ To investigate the zooplankton metabolic responses, we performed zooplankton community gene expression (metatranscriptomic) analysis across eutrophic and mesotrophic environments. We focus on analyzing the gene annotated to Kyoto Encyclopaedia of Genes and Genomes (KEGG) as this database provides the most complete metabolism functional annotation for eukaryotic organisms.^77^ Briefly, functional annotation of these zooplankton community-level transcripts identified 12,663 transcripts with KEGG orthologs, allowing us to explore trophic-driven metabolic variation. Additionally, taxonomic classification of these metatranscriptomes revealed 19 zooplankton orders (Supporting Information Figure S6), representing approximately 80% of total relative abundance, while the remaining taxa were annotated to the phylum or class level. The dominant taxa across all regions include copepods such as *Calanoida, Harpacticoida*, and *Siphonostomatoida* as well as *Diplostraca* and *Decapoda.*

First, we examined the genes annotated with KEGG metabolism subcategories to investigate the zooplankton metabolic responses toward different environmental parameters. Energy metabolism gene expressions showed strong positive correlations with phosphate (PO_4_^3–^) and ammonium (NH_4_^+^) concentrations (Figure 3a), indicating that zooplankton in eutrophic environments would enhance energy production to fuel biosynthetic demands. This process may explain the increase in phosphorus- and nitrogen-containing-derived DOM release, such as protein and amino sugar-like compound derivatives, under eutrophic conditions, as shown in Figure 2d. Similarly, the positive association between lipid metabolism genes and NH_4_^+^ suggests increased lipid processing in eutrophic environments with potential downstream effects on lipid-like DOM exudation. This occurs likely because NH_4_^+^ concentration may influence phytoplankton composition, providing zooplankton with lipid-rich phytoplankton prey.^78^ As zooplankton consume these lipid-dense resources, their metabolic demands may shift toward higher lipid anabolism to support molting or reproduction (such as for gonad development) processes.^79^ We also observed that genes associated with carbohydrate metabolism and glycan biosynthesis exhibited negative correlations with temperature. This temperature effect may arise because zooplankton in warmer waters may shift energy allocation away from complex carbohydrate metabolism toward other essential processes for survival such as enhanced respiration and ATP production to meet increased metabolic demands.^80,81^ Furthermore, the negative correlations between dissolved oxygen (DO) and genes involved in energy and amino acid metabolism suggest that zooplankton do not need to elevate their metabolic activity when oxygen availability is high. Adequate oxygen already supports respiration^82^ and aerobic processes involved in amino acid metabolism^83^ (Figure 3a).

**Figure 3:**
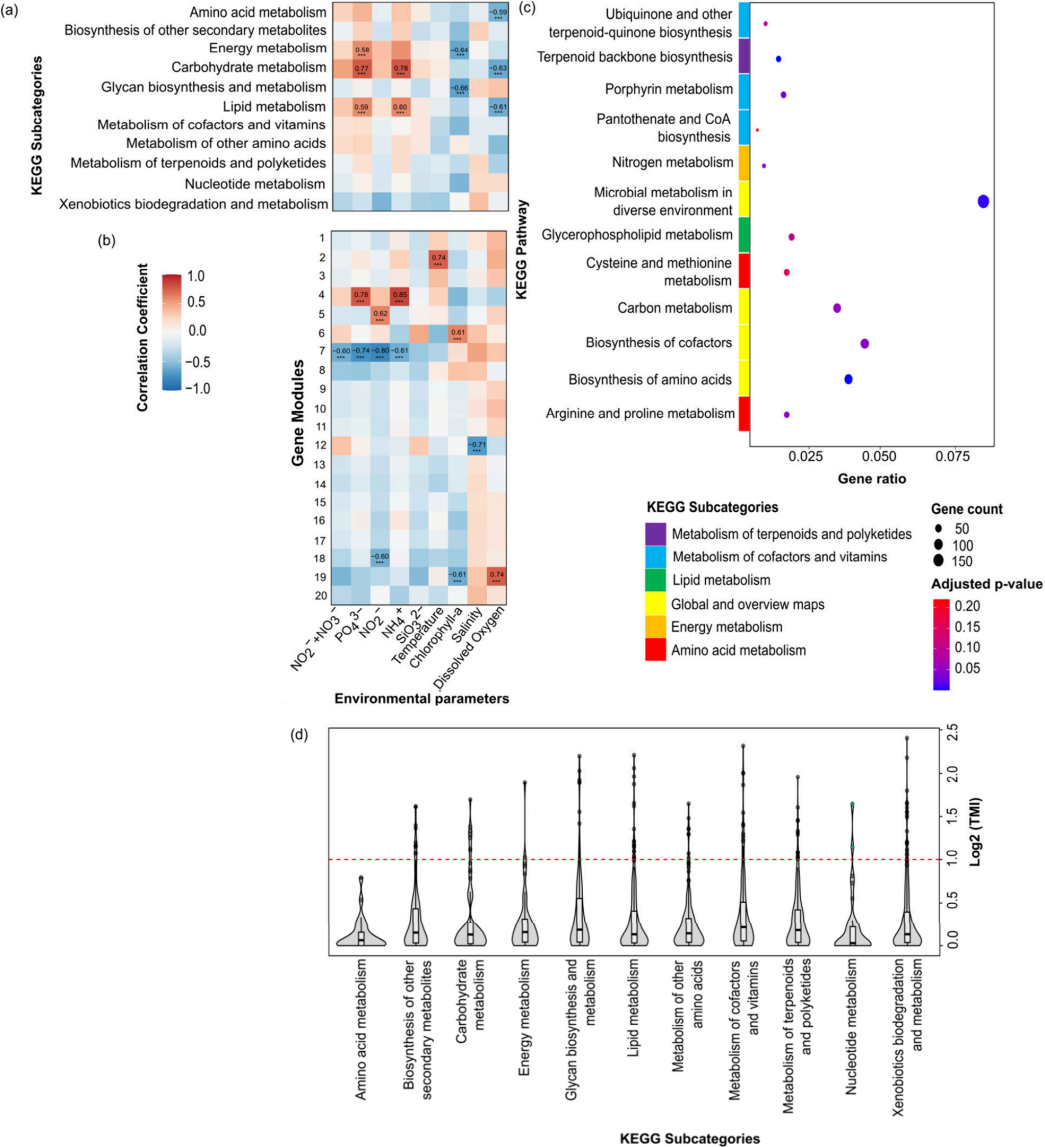
(a) Correlation between environmental parameters (*x*-axis) and KEGG metabolism subcategories (*y*-axis). The top numbers in each cell represent Pearson’s correlation coefficients, while the bottom asterisk indicates the significant p value (*** = p value <0.001) from the correlation test. (b) Weighted gene coexpression network analysis illustrates the correlation between environmental parameters (*x*-axis) and groups of similarly expressed genes, assigned to gene modules (ME) (*y*-axis). The top numbers in each cell represent Pearson’s correlation coefficients, while the bottom numbers indicate the p-values from the correlation test. The color of each cell reflects the strength of the correlation between the ME and environmental parameters, with red indicating a positive correlation and blue indicating a negative correlation between environmental parameters (*x*-axis) and KEGG metabolism subcategories (*y*-axis). (c) Dot plot of KEGG enrichment analysis for module 4. Enrichment is calculated based on the number of KOs overrepresented in KEGG pathways compared to all annotated KOs. Significance is determined using false discovery rate-adjusted p-values of <0.05, with darker colors indicating higher significance. The gene count represents the number of KOs detected in each pathway, and different color annotations correspond to KEGG subcategories. (d) Ratios of KEGG subcategory profiles between eutrophic and mesotrophic nutrient regions (TMI). The red dashed line indicates a ratio of 1; values below 1 signify lower expression of metabolic gene functions in eutrophic regions relative to mesotrophic regions, while values above 1 indicate the opposite.

To further complement our previous gene-by-gene analysis results, we aimed to identify specific gene groups in zooplankton communities that exhibit coordinated expression patterns linked to environmental parameters. We employed an established weighted gene coexpression network analysis (WGCNA) to KEGG-annotated genes. This method clusters genes into modules based on highly correlated expression patterns across samples, allowing us to reveal potential gene networks associated with specific environmental conditions that previous gene-by-gene analyses may have overlooked.^66,84^ We identified 20 distinct coexpression modules (Figure 3b). Among these, modules 4, 5, 7, and 18 demonstrated significantly high correlations with nutrient concentrations including NO_3_^–^, N_2_O^–^, PO_4_^3–^, SiO_3_^2–^, and NH_4_^+^. We then performed gene enrichment analysis on those modules to identify the key gene members of each module. We found that modules 4 and 7 have key gene members related to metabolic processes; therefore, we focus on these two.

Module 4 positively correlates with nutrient level (PO_4_^3–^), exhibiting enrichment in KEGG pathways associated with amino acid metabolism, energy metabolism, cofactor-vitamin biosynthesis, and terpenoid-polyketide metabolism (Figure 3c). These metabolic processes may support the release of nitrogen- and phosphorus-rich DOM compounds such as protein derivatives, as reported earlier in Figure 2. In contrast, module 7 (Supporting Information Table S3), negatively associated with nutrient levels, featured pathways related to retinol metabolism, cytochrome P450-mediated detoxification, and linoleic acid metabolism, reflecting increased lipid turnover and oxidative stress responses under mesotrophic conditions.^85–89^ These coexpression gene patterns highlight how zooplankton communities actively modulate specific metabolic pathways such as energy optimization under nutrient scarcity^90^ — in response to shifting trophic conditions. The results further underscore nutrient availability as a key driver of zooplankton community metabolic plasticity.

Considering that nutrient concentrations are the dominant environmental parameters contrasting the sampling sites (Figure 1b), we continue to examine the zooplankton gene expressions by comparing their metabolic profiles across trophic gradients directly. Unlike the previous results, which focused on KEGG metabolism associations with environmental parameters, this analysis provides a specific comparison of zooplankton metabolic activities across eutrophic and mesotrophic environments. To achieve this, we developed a quantification approach (see Materials and Methods for details) to systematically study the KEGG metabolic pathways by dividing the relative expression of KEGG-annotated metabolic genes between zooplankton communities in eutrophic and mesotrophic environments. We named this approach the transcriptomic metabolic index (TMI), inspired by the transcriptomic age index.^91^ This TMI is a complementary, systems-level metric intended to summarize aggregate metabolic gene expression rather than identify individual differentially expressed genes. Interestingly, in all KEGG metabolism categories, zooplankton in eutrophic regions exhibited lower expression of metabolism-related genes compared to their mesotrophic counterparts (Figure 3d). This pattern reflects fundamental physiological differences driven by strategic resource allocation in zooplankton.^85,92,93^ Mesotrophic populations, facing nutrient scarcity, broadly upregulate metabolic processes critical for nutrient acquisition, stoichiometric homeostasis, and survival, including enhanced biosynthesis of cofactors, energy-efficient substrate utilization, and biomolecule recycling. This response to nutrient scarcity necessitates higher gene expression across diverse metabolic pathways to maximize resource extraction from low-nutrient prey.^85,92,94^ In contrast, abundant nutrients in eutrophic environments may alleviate the pressure for stringent metabolic regulation and optimization of nutrient uptake.^92^ This allows for metabolic streamlining, such as the suppression of nonessential biosynthetic pathways, reducing overall transcriptional overhead while selectively upregulating specific processes crucial for exploiting nutrient abundance.^92,95,96^

### Zooplankton-Derived DOM Chemical Properties Correlate with Metabolic Gene Functions

In the previous sections, we described the differences in zooplankton-derived DOM characteristics and metabolic responses across trophic gradients. Given that zooplankton-derived DOM may originate from organismal metabolic outputs,^4^ we then sought to investigate how internal metabolic processes may regulate the chemical properties of the derived DOM. To do so, we conducted two Spearman correlation analyses: one examining the relationship between DOM molecular composition and metabolic gene function (Figure 4a), and another assessing the correlation between DOM molecular traits and metabolic gene function (Figure 4c). The first Spearman correlation revealed significant linkages between DOM molecular composition and gene function (Figure 4a). Specifically, unsaturated hydrocarbons showed positive correlations with amino acid and terpenoid-polyketide metabolism gene functions. In contrast, protein-like DOM molecules exhibited negative associations with carbohydrate, energy, and lipid metabolism genes. This negative correlation aligns with the reduced transcriptional investment in the three groups of metabolic genes observed in eutrophic zooplankton communities (Figure 3d). When nutrient are abundant, zooplankton likely allocate fewer metabolic processes for prey’s protein turnover as their nutrient needs are sufficient.^97^ In contrast, zooplankton in mesotrophic environments broadly upregulate metabolic pathways^92,94^ (Figure 3d) to compensate for lower prey abundance and nutritional quality.^98,99^ As a result, more protein is retained for metabolic needs, which can reduce the release of protein-like DOM.

**Figure 4:**
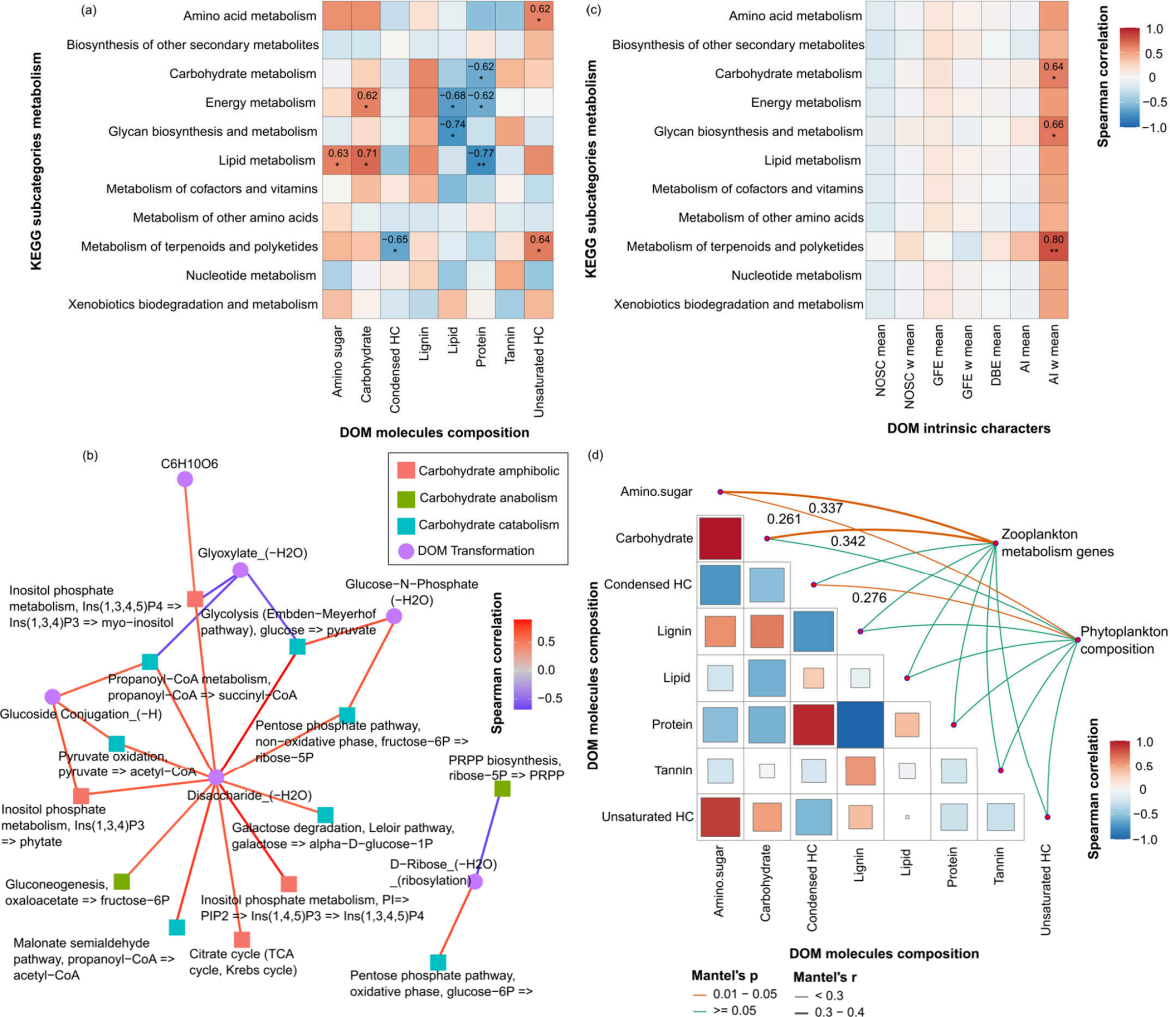
Association between DOM chemical properties, zooplankton metabolic gene functions, and phytoplankton composition. (a) Spearman’s rank correlations between zooplankton’s KEGG subcategories metabolism and the composition of DOM molecules. Significant correlations (* = p value <0.05, ** = p value <0.01) are indicated by the asterisks, with correlation coefficients shown within each cell. (b) Association between carbohydrate metabolism genes and carbohydrate-like (sugar) DOM transformations. Square nodes represent carbohydrate metabolism genes, with node colors indicating metabolic categories (anabolic, catabolic, or amphibolic). Circle nodes represent individual DOM transformations. Edge colors denote the correlation strengths between gene expression and DOM transformation frequencies. (c) Spearman’s rank correlations between zooplankton’s KEGG subcategories metabolism and DOM intrinsic characters, including the nominal oxidation state of carbon (NOSC), Gibbs energies of the oxidation half reactions (Δ*G*_*Cox*_^0^), modified aromaticity index (AImod), and double bond equivalent (DBE). Significant correlations (* = p value <0.05, ** = p value <0.01) are indicated by the asterisks, with correlation coefficients shown within each cell. (d) Mantel test results showing relationships between DOM molecular classes, zooplankton metabolic gene profiles, and phytoplankton community composition.

As heterotrophic grazers, zooplankton obtain essential nutrients by feeding on phytoplankton and other primary producers.^4,12^ During ingestion and growth, they assimilate and metabolize organic matter from their prey through enzymatic reactions.^92,100^ Consequently, the molecular composition of zooplankton-derived DOM can be shaped predominantly by either 1) passive dietary inputs,^101^ 2) active internal metabolic regulation, or 3) both.^97^ If dietary input was the main driver of zooplankton-derived DOM composition, we would expect lower expression of amino acid catabolic pathways under mesotrophic conditions. This is because prey with lower nutrient content would lead to reduced activity of protein degradation.^92,97,98,102^ Instead, we observed the opposite pattern; the amino acid catabolic pathways showed higher expression in mesotrophic than in eutrophic communities (Supporting Information Figure S7). This higher amino acid catabolic pathway, rather than being a passive reflection of lower dietary protein, suggests actively upregulation of protein degradation to fulfill the zooplankton metabolic demands^97^ (“you are how you process what you eat”). In addition, amino acid anabolic pathways also exhibit higher expression in mesotrophic environment, which is consistent with metabolic compensation response to the limited prey’s nutrient content.^92,94^ Therefore, the expression patterns of both catabolic and anabolic gene pathways suggest that internal metabolic regulation, as a main driver, are actively influencing the molecular composition of zooplankton-derived DOM.

Furthermore, we observed negative correlations between lipid-like DOM molecules and both energy metabolism as well as glycan biosynthesis pathways. This relationship suggests that in eutrophic environments, zooplankton prioritize energy-efficient metabolic pathways, such as carbohydrate rather than lipid metabolism.^58,73^ As a result, their release of lipid-like DOM becomes lower. The relationship can arise because high nutrient availability may facilitate streamlined metabolic processes, reducing the necessity for extensive lipid catabolism.^93,97,104^ The condensed hydrocarbon-like molecules were also negatively correlated to terpenoid-polyketide metabolism genes, which indicates the pathways responsible for synthesizing complex hydrocarbons are downregulated in eutrophic zooplankton communities. Lastly, the positive correlations between carbohydrate and amino sugar-like DOM compounds and the expression of energy and lipid metabolism genes further illustrate that the release of carbohydrate and amino sugar-like DOM is likely driven by zooplankton metabolic processes to meet their energy needs.^93,104–106^

To further examine the mechanistic links between zooplankton metabolic processes and their derived DOM molecules, we calculated correlation between zooplankton metabolic gene functions and DOM molecular transformations (Figure 4b). Several pathways involved in the central carbon metabolism, such as glycolysis, TCA cycle, pyruvate oxidation, and the malonate semialdehyde pathways,^105^ showed high positive correlations (r = 0.69–0.90; p value <0.05) with disaccharide-related transformations (Disaccharide (−H_2_O)). Likewise, both the oxidative and nonoxidative phases of the pentose phosphate pathway exhibited substantial positive associations (r = 0.66–0.71; p value <0.05). These patterns collectively may happen because carbohydrate metabolic pathways can directly influence biosynthetic fluxes derived from sugar substrates, including disaccharide.^105,107,108^ Inositol phosphate metabolism (e.g., Ins­(1,3,4,5)­P_4_ → Ins­(1,3,4)­P_3_ → myo-inositol) was also positively correlated with tentative disaccharide and hexose-related transformations (r = 0.56–0.88; p value <0.05), suggesting that regulatory and signaling pathways^109,110^ may contribute additional influences on carbohydrate-like molecular class. Moreover, the strong correlations with sugar-like transformations may imply that shifts in zooplankton cellular regulation can manifest directly in the chemical composition of their released DOM. The involvement of galactose degradation (galactose → αDglucose1P; r = 0.61; p value <0.05) may further reinforce the central role of carbohydrate interconversion^94^ in shaping DOM molecular patterns.

We also observed that ribosylation transformations (D-Ribose (−H_2_O)) displayed contrasting correlations across pathways. PRPP biosynthesis (ribose5P → PRPP) showed a negative association (r = – 0.68; p value <0.05), whereas the oxidative branch of the pentose phosphate pathway (glucose6P → ribulose5P) exhibited a strong positive correlation (r = 0.71; p value <0.05). These opposing patterns likely reflect a balance between anabolic nucleotide precursor formation and catabolic pentose generation.^111,112^ Such metabolic trade-offs appear to leave distinct molecular imprints on ribose-related DOM transformations. We additionally detected negative correlations (r = – 0.62 to – 0.68; p value <0.05) between glyoxylate-related transformations (Glyoxylate (−H_2_O)) and pathways such as glycolysis, inositol phosphate metabolism, and propanoyl-CoA metabolism. The glyoxylate cycle is primarily observed in plants, bacteria, protists, fungi, and nematodes for the conversion of acetate into carbohydrates. Meanwhile, metazoans generally lack a canonical glyoxylate cycle. Thus, any glyoxylate-like transformation may come from the undigested prey.^113–115^ Despite the PMD approach we used can only infer putative transformations due to the structural complexity of DOM, it remains a powerful framework for predicting transformation patterns.^57–60^

The last Spearman correlation analysis showed that zooplankton metabolic processes emerged as a potential key driver of DOM intrinsic characters (Figure 4c). This was evident from the strong positive correlation among particular gene functions, such as amino acid, carbohydrate, and terpenoid/polyketide metabolism, with the modified aromaticity index weighted average (AI w mean) of the derived DOM. This correlation may imply a mechanistic link, and upregulation of certain metabolic processes may enhance biosynthesis of higher aromatic metabolites. The aromaticity characters of DOM critically influence its bioavailability by modulating interactions with microorganisms and chemicals in the environment.^116^ Higher aromatic content generally corresponds to stronger sorption affinity and altered microbial community responses and can decrease or modify bioavailability of various substances such as pollutants or nutrients.^117^

To further disentangle whether zooplankton-derived DOM is shaped predominantly by dietary input or by how they metabolically process those inputs (metabolic regulation), we evaluated how strongly each factor correlates with DOM composition. Specifically, we applied a Mantel test integrating DOM molecular classes, zooplankton metabolic gene expression profiles, and phytoplankton community composition (used as a proxy for dietary input). The result revealed that zooplankton metabolic gene expressions exert a higher association with zooplankton-derived DOM rather than phytoplankton composition (Figure 4d). Among the DOM molecular classes, carbohydrate-like and amino sugar-like compounds showed stronger correlations with zooplankton metabolic gene profiles. While we also observed phytoplankton taxonomic composition association with condensed hydrocarbon-like DOM, the mantel r value was lower. Thus, rather than viewing the mechanisms of “you exudate what you eat”^101^ and “you are how you process what you eat” as mutually exclusive, our results suggest that they act in concert to determine the molecular profiles of zooplankton-derived DOM, with metabolic regulation acting as the dominant one.

Taken together, our results provide novel insights that zooplankton metabolic activity has a strong link to the molecular composition and aromaticity characteristics of DOM they release. This mechanistic link may explain the mechanism behind the distinct pattern of zooplankton-derived DOM molecular properties across the nutrient gradient. Situating these results within the broader DOM literature reveals that most prior work has focused on different stages of the DOM cycle. Previous studies have primarily examined DOM transformation mediated by an arsenal of enzymes such as laccases,^118^ esterase,^119^ and peroxidases^120^ associated with microbial metabolism. However, these studies focus on degradation or transformation processes of DOM by microbial activities.^118–122^ In addition, while several studies have reported that zooplankton contributed to the release of complex DOM molecules,^17,57,75^ they have not linked these compounds to the internal metabolic regulation of zooplankton communities. By linking zooplankton metabolic activity to the molecular properties of their derived DOM, our study offers a complementary perspective and reveals a previously overlooked mechanism.

### Implications

Our results show differences in zooplankton-derived lipid, protein, and unsaturated hydrocarbon-like DOM molecules, as well as variations in their aromaticity signatures between mesotrophic and eutrophic environments. Furthermore, these chemical properties of DOM were significantly correlated with zooplankton metabolic gene functions. By bridging metatranscriptome and DOM chemical properties, we show that metabolic gene expression is not just showing the organism’s physiological response but can be a blueprint for DOM generation. The strong association between DOM molecular properties and metabolic genes demonstrates that gene expression can serve as a candidate proxy for predicting organism-derived DOM characteristics. This may open new avenues for studying DOM dynamics in aquatic systems (Figure 5).

**Figure 5:**
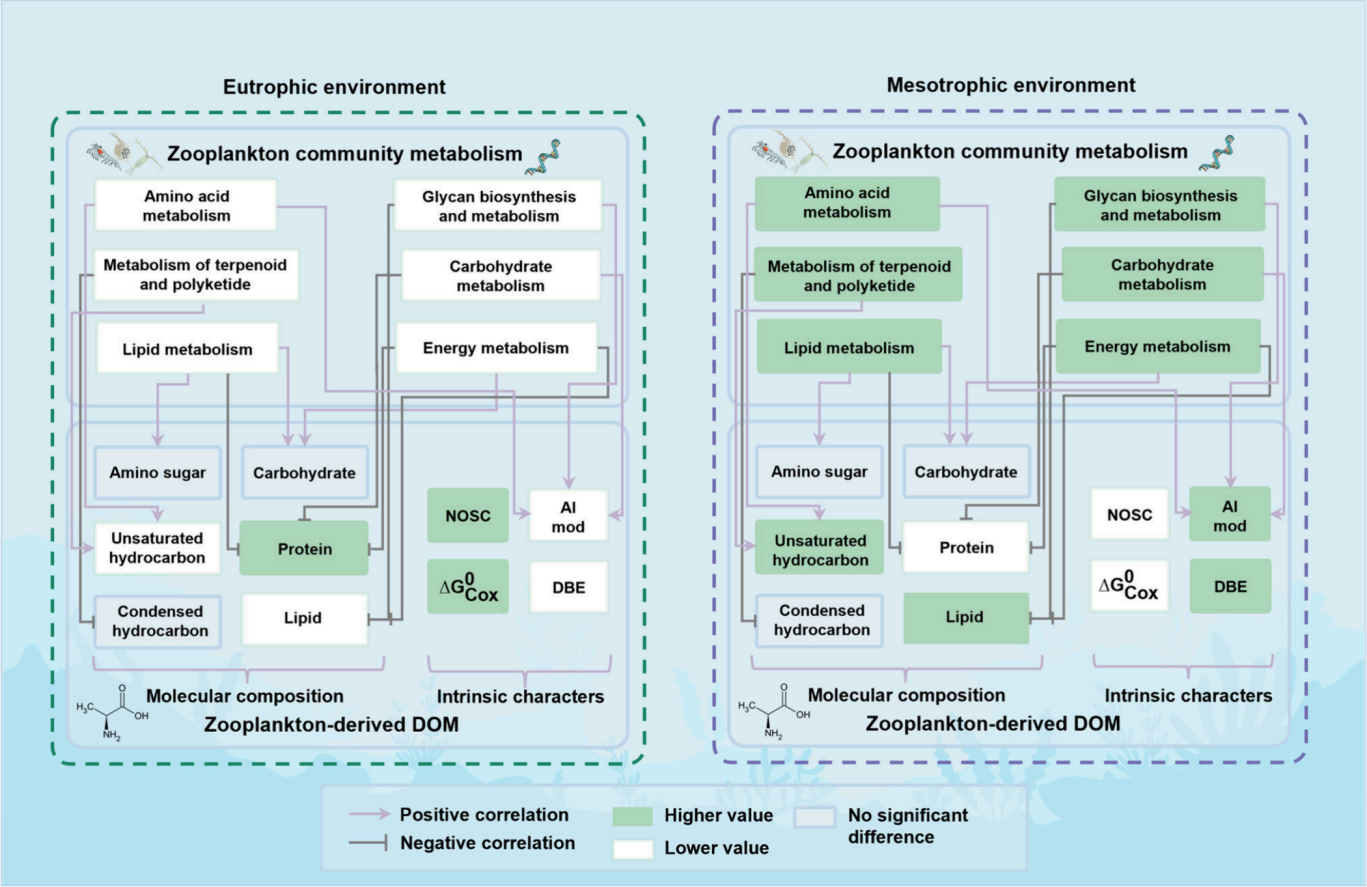
Conceptual diagram illustrating the role of zooplankton metabolism in the aquatic dissolved organic matter (DOM) cycle. Zooplankton communities inhabiting mesotrophic environments exhibit higher expression of metabolic gene functions than those in eutrophic regions. These metabolic adjustments will modulate the molecular composition and properties of the zooplankton-derived DOM.

While our study provides valuable insights, it is important to consider certain limitations. Our operational definition of zooplankton-derived DOM, based on a subtractive comparison between incubation and paired controls, was intentionally designed to ensure that the molecules reported are unequivocally linked to active zooplankton metabolism during the experiment. By focusing exclusively on compounds that appeared in the incubation treatments but were absent from the corresponding controls, we minimized interference from other autochthonous DOM sources (e.g., phytoplankton-derived materials). This subtraction method may underestimate the total contribution of the zooplankton to the ambient DOM pool. Nevertheless, it provides a conservative and highly specific characterization of newly released DOM compounds associated with the zooplankton activity. Moreover, this approach aligns with previous studies of zooplankton-derived DOM, which utilize natural seawater as the control.^23^ Future studies incorporating timeseries sampling or isotopic labeling may complement this framework by distinguishing between preexisting and newly produced zooplankton-derived DOM under natural conditions. Additionally, our study utilized an incubation set up in static water, which differs from the dynamic conditions of the open ocean, where released DOM compounds would dissipate through diffusion and advection. Thus, the observed zooplankton-derived DOM should be interpreted as offering mechanistic insights rather than representing absolute concentrations in situ. Moreover, future studies should prioritize validating gene expression-DOM relationships in controlled laboratory experiments and expanding metabolism gene annotation in eukaryotic organisms. This will ultimately support the development of models capable of predicting how changes in zooplankton metabolic activity influence the DOM molecular composition under varying environmental conditions. To sum up, our study provides evidence of how zooplankton metabolic processes play roles in DOM cycling by modulating derived DOM molecular properties. This modulation is driven by the metabolic plasticity of the zooplankton community in response to nutrient availability.

## Supplementary Material



## Data Availability

The molecular formula data from the FT-ICR MS measurement results presented in this paper and the molecular transformation database used to calculate the putative DOM molecular reactions are openly available through Zenodo at 10.5281/zenodo.17156986. The zooplankton raw sequence data reported in this paper are available in NCBI BioProject and SRA Accession number: PRJNA1338574.

## Notes

**Text S1.** Trophic state index calculation method; **Text S2.** Phytoplankton community composition analysis; **Figure S1.** Schematic diagram of the zooplankton sampling, DOM release incubation, zooplankton RNA processing, and subsequent characterization of derived DOM using FT-ICR MS; **Figure S2.** Total dissolved phosphate concentration; **Figure S3.** Principal component analysis of the DOM molecular formulas; **Figure S4.** Comparison between zooplankton and three representative phytoplankton-derived DOM molecular classes compositions; **Figure S5.** Relative abundance of phytoplankton (genus level) comparison between eutrophic and mesotrophic regions; **Figure S6.** Relative Abundance (%) of the zooplankton community at the order level in Eutrophic and Mesotrophic regions. **Figure S7.** Comparison of amino acid biosynthesis and degradation gene expression between mesotrophic and eutrophic zooplankton communities; **Table S1.** Environmental parameters in each sampling station; **Table** **S2.** DOM molecular index calculation formula; **Table S3.** Significantly enriched gene members of module 7 (one of the representative modules from the WGCNA analysis) (PDF)
