# Supplementary material for: Zooplankton Metabolism Shapes Molecular Composition of Dissolved Organic Matter in Coastal Waters
Source: Environ Sci Technol. 2026 May 6;60(22):15873–87. doi: 10.1021/acs.est.6c05592 (PMC13262054; doi:10.1021/acs.est.6c05592)
Supplement: Supplementary file 1 [file es6c05592_si_001.pdf]

Zooplankton Metabolism Shapes Molecular Composition of Dissolved Organic Matter in Coastal Waters

Muhammad Firman Nuruddin[1,2], Poon Yiu Cho Joe[1], Xuejia He[3], Zekun Zhang[1], Xiaoming Tang[4], Sangwook Scott Lee[1], Shuwen Zhang[4], Ding He[1,2], Longjun Wu[1,2*]

[1]Department of Ocean Science, The Hong Kong University of Science and Technology, Hong Kong SAR, China.

[2]Center for Ocean Research in Hong Kong and Macau, Hong Kong SAR, China.

[3]College of Life Science and Technology, Key Laboratory of Eutrophication and Red Tide Prevention of Guangdong Higher Education Institutes, Jinan University, Guangzhou 510632, Guangdong, China

[4]Guangdong Provincial Key Laboratory of Biotechnology for Plant Development, Guangzhou Key Laboratory of Subtropical Biodiversity and Biomonitoring, South China Normal University, West 55 of Zhongshan Avenue, Guangzhou, 510631, PR China

Table of contents

Text S1. Trophic state indexes calculation method..3

Text S2. Phytoplankton community composition analysis 4

References 15

Table of figures

Figure S1. Schematic diagram of the zooplankton sampling, DOM release incubation, zooplankton RNA processing, and subsequent characterization of derived DOM using FT-ICR MS 5

Figure S2. Total dissolved phosphate concentration 6

Figure S3. Principal component analysis of the DOM molecular formulae 7

Figure S4. Comparison between zooplankton and three representative phytoplankton-derived DOM molecular classes compositions 8

Figure S5. Relative abundance of phytoplankton (genus level) comparison between eutrophic and mesotrophic regions 9

Figure S6. Relative Abundance (%) of the zooplankton community at the order level in (a) Eutrophic and (b) Mesotrophic regions...10

Figure S7. Comparison of amino acid biosynthesis and degradation gene expression between mesotrophic and eutrophic zooplankton community 11

Table of Tables

Table S1. Environmental parameters in each sampling stations.....................................12

Table S2. DOM molecular indexes calculation formula 13

Table S3. Significantly enriched gene members of module 7 (one of the representative modules from the WGCNA analysis)..14

Text S1. Trophic state indexes calculation method

To calculate Trophic state indexes (TRIX) we use the equation below[1]:

$$\mathrm{TRIX} = \frac{\log_{10}[(\mathrm{DIP} \times \mathrm{DIN} \times \mathrm{Chla} \times \mathrm{DO}) + \mathrm{a}]}{\mathrm{b}}$$

Where:

(1) DIP: Dissolved inorganic phosphorus (mg. L^{-1}).

(2) DIN: Dissolved inorganic nitrogen (mg. L^{-1}).

(3) Chl*a*: Chlorophyll-*a* concentration (mg. m^{-3}).

(4) DO: The % deviation of the oxygen concentration from saturation conditions.

(5) a,b: a = 1.5 and b = 1.2 are scale coefficients[2] to fix the lower limit value of the index and also to fix the scale range from 2 to 10.

(6) $\times$: multiplication

The sampling sites were classified as mesotrophic when the TRIX trophic index values ranged from five to six, while sites were classified as eutrophic when the index value exceeded seven[3]. During the calculations, none of the variables were below the detection limit. However, if such a case occurs, an imputed value—set lower than the lowest measured concentration—may be used to avoid zero values in the TRIX computation.

Text S2. Phytoplankton community composition analysis

To characterize the phytoplankton communities, 100 mL of subsamples seawater were fixed with acidic Lugol's solution (to a final concentration of 2%) and stored in amber plastic bottles at room temperature. Subsamples of 10 mL were counted after settling for 24 h in sedimentation chambers following Utermöhl (1958)[4] and observed under an Olympus IX51 inverted microscope with a magnification of 200 or 400 ×. At least 50 cells were counted for each sample.[5]

To characterize the phytoplankton communities, 1 L seawater subsamples were collected from the surface layer and immediately fixed with acidic Lugol's iodine solution to a final concentration of 2% (v/v). Samples were stored in amber plastic bottles at room temperature and analyzed within 6 months.

For quantitative analysis, fixed samples were allowed to settle for a minimum of 24 h. Following sedimentation, the supernatant was carefully siphoned off using a 6 mm diameter silicone hose fitted with a 20 μm mesh sleeve at the outlet to prevent resuspension or loss of settled cells. This siphoning procedure was repeated gradually until the remaining sample volume was reduced to approximately 10-50 mL. The concentrated residue was then thoroughly homogenized to ensure even cell distribution prior to microscopic examination. Phytoplankton cells were enumerated using a 0.1 mL plankton counting chamber under an Olympus BX-53 inverted microscope at magnifications of 200× or 400×, depending on cell size. Taxonomic identification was conducted to the genus or species level based on standard identification keys and descriptions provided by Jin et al. (1965)[6], Yang & Dong (2006)[7], and Lü et al. (2024)[8]. The abundance of each taxonomic group was recorded accordingly. A minimum of 100 cells per sample were counted to ensure quantitative accuracy and reproducibility.

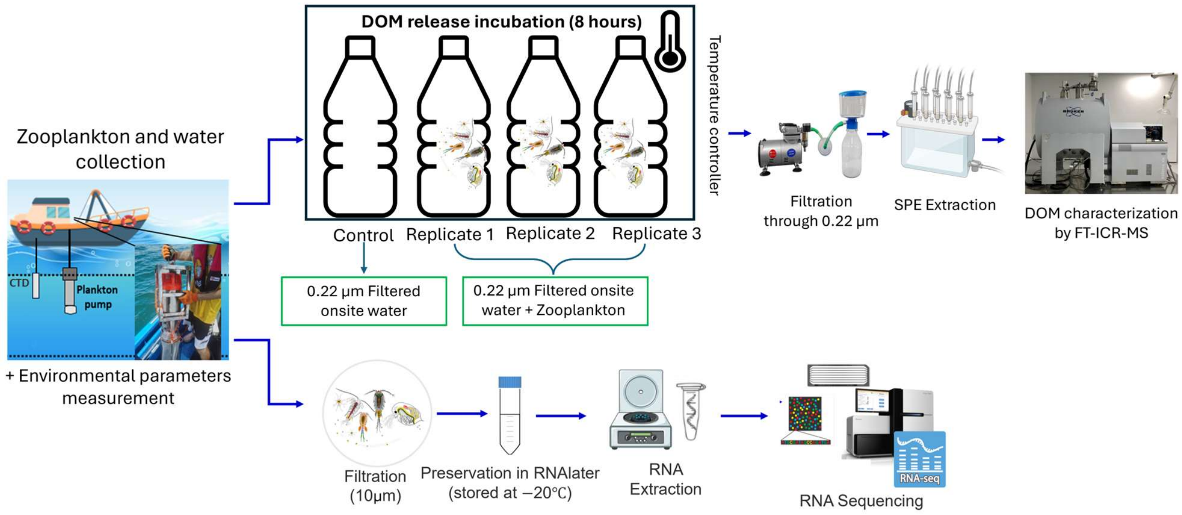

Figure S1. Schematic diagram of the zooplankton sampling, DOM release incubation, zooplankton RNA processing, and subsequent characterization of derived DOM using FT-ICR MS.

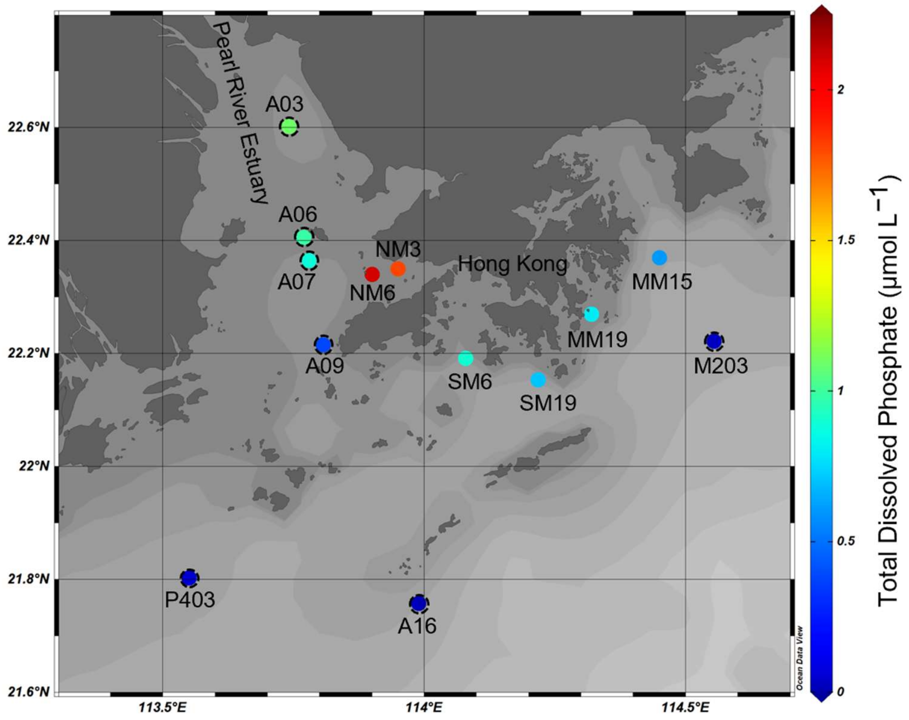

Figure S2. Total dissolved phosphate concentration (μmol L^{-1}). The stations for zooplankton derived DOM release incubation were indicated by dashed circles. The station naming codes are as follows: A, M, and P refer to stations along transects A, M, and P during the AOE Cruise, respectively; NM, MM, and SM denote stations in the Northern, Mirs Bay, and Southern Hong Kong monitoring regions, respectively.

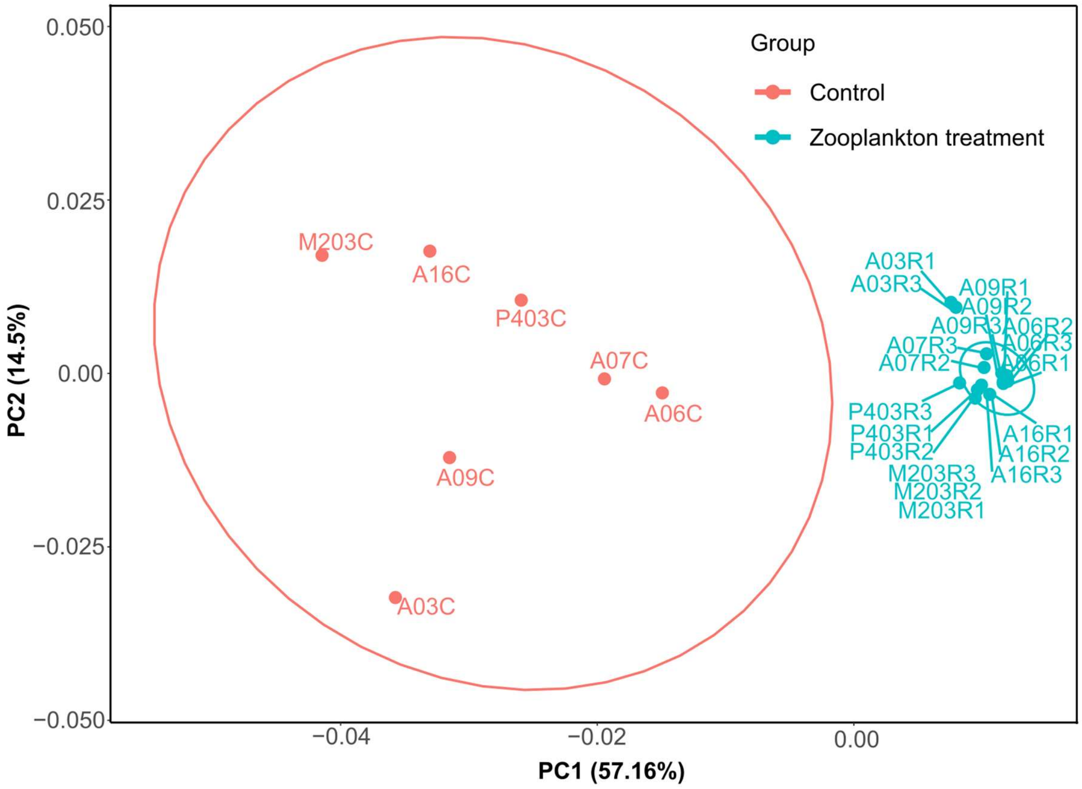

Figure S3. Principal component analysis of the DOM molecular formulae. The dots represent sampling stations, and the colors denote treatment groups (control and zooplankton treatment).

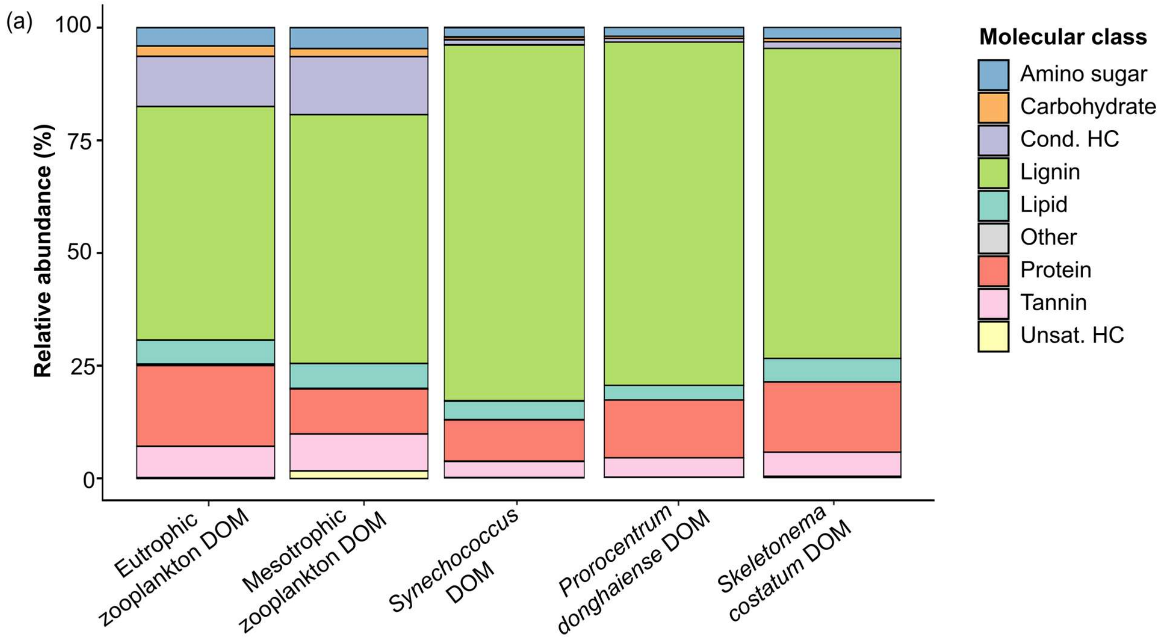

Figure S4. Comparison between zooplankton and three representative phytoplankton (cyanobacteria *Synechococcus sp.,* diatom *Prorocentrum donghaiense*, and dinoflagellate *Skeletonema costatum*) derived DOM molecular classes compositions.

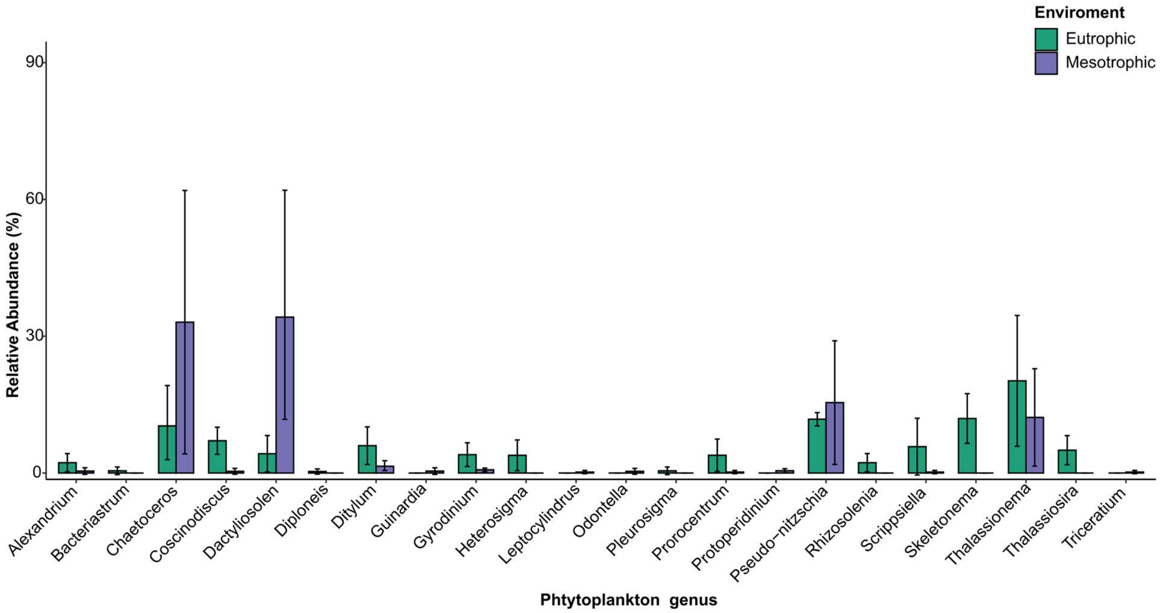

Figure S5. Relative abundance of phytoplankton (genus level) comparison between eutrophic and mesotrophic regions. The colours represent samples regions, and the error bars represents standard deviations.

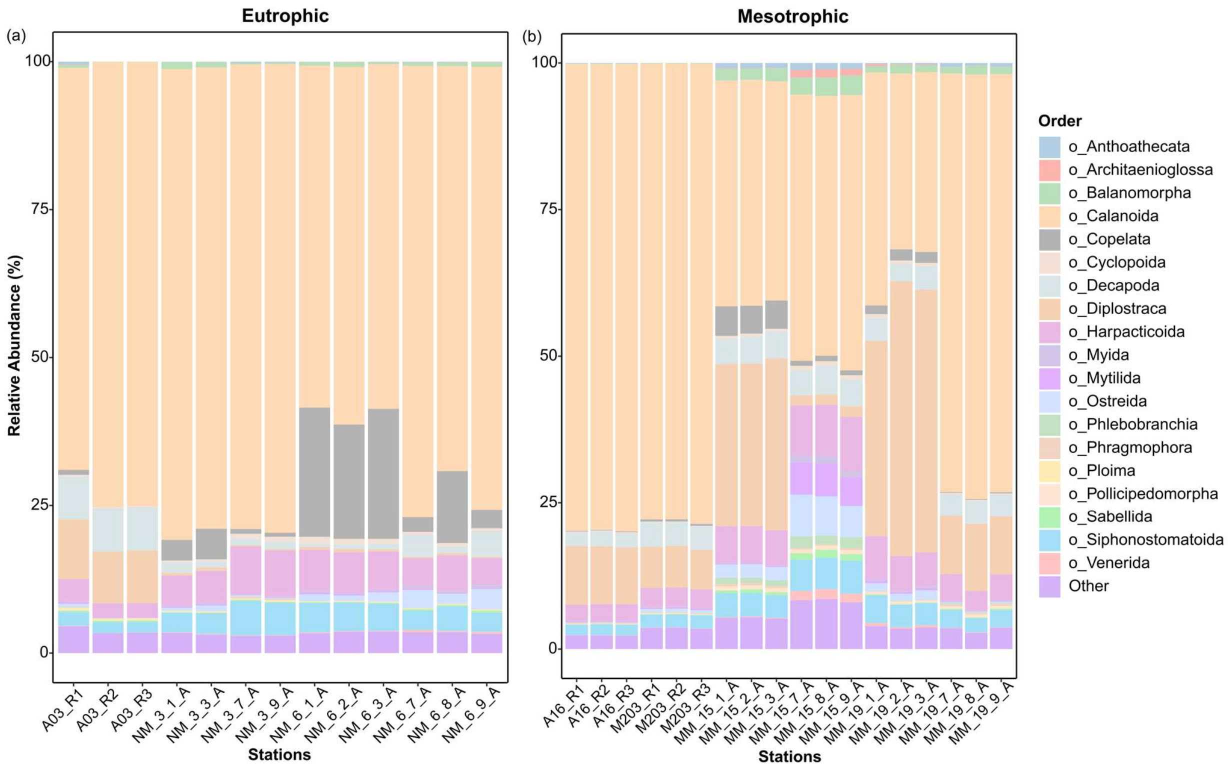

Figure S6. Relative Abundance (%) of the zooplankton community at the order level in (a) eutrophic and (b) mesotrophic regions. The colours represent zooplankton orders. The station naming codes are as follows: A and M refer to stations along transects A and M during the AOE Cruise, respectively; NM and MM denote stations in the Northern Hong Kong and Mirs Bay monitoring regions, respectively.

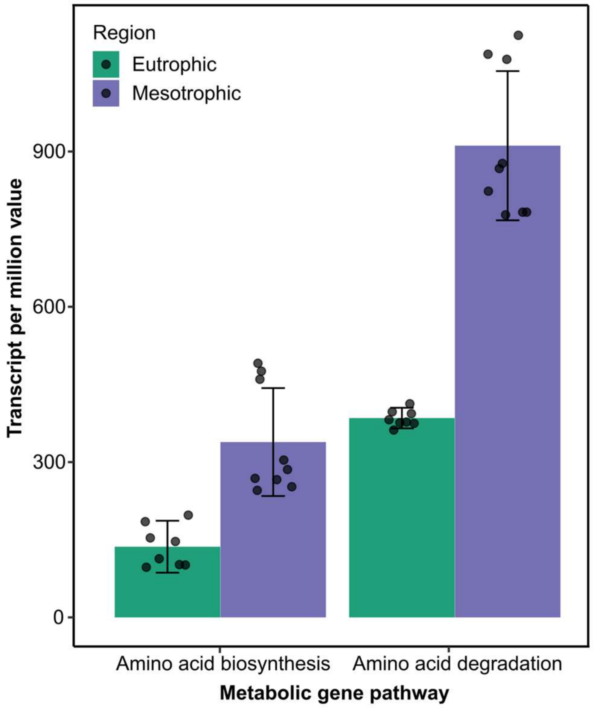

Figure S7. Comparison of amino acid biosynthesis and degradation gene expression between mesotrophic and eutrophic zooplankton community. The bar colours represent the different regions, the dots denote individual stations, and the error bars represents standard deviations.

Table S1. Environmental parameters in each sampling stations. The station naming codes are as follows: A, M, and P refer to stations along transects A, M, and P during the AOE Cruise, respectively; NM, MM, and SM denote stations in the Northern, Mirs Bay, and Southern Hong Kong monitoring regions, respectively.

Station	Total Dissolved Nitrogen (μmol L^{-1})	Total Dissolved Phosphorous (μmol L^{-1})	NO_3^- (μmol L^{-1})	NO_2^- (μmol L^{-1})	Ammonium (μmol L^{-1})	Silicate (μmol L^{-1})	Chlorophyll-a (μg L^{-1})	Temperature (°C)	Salinity (psu)	Dissolved Oxygen (μg L^{-1})
A03	104.88	1.09	102.98	1.89	0.75	104.01	5.69	29.56	1.05	5.55
A16	0.63	0.01	0.61	0.02	0.10	3.54	1.71	31.34	31.87	6.68
M203	2.08	0.02	1.62	0.46	0.25	0.91	7.49	30.10	33.33	6.33
MM15	13.02	0.60	12.98	0.04	0.93	4.03	1.78	28.29	31.38	6.30
MM19	27.03	0.80	26.90	0.13	1.36	4.98	6.80	27.57	31.24	6.88
NM3	70.49	1.80	67.60	2.89	4.05	40.67	6.70	28.23	22.10	5.42
NM6	90.16	2.10	86.73	3.43	4.51	52.01	11.60	28.75	18.81	5.85
SM19	28.73	0.70	28.09	0.64	1.65	7.94	7.62	28.50	27.73	7.35
SM6	35.89	0.90	34.95	0.94	2.14	9.75	13.81	28.50	26.55	7.52
P403	1.01	0.05	1.00	0.01	0.24	9.36	3.89	29.86	31.02	6.08
A06	89.08	0.98	85.82	3.26	0.02	105.16	3.70	29.14	6.98	6.03
A07	88.66	0.88	85.70	2.96	0.02	98.59	4.43	28.80	9.19	5.77
A09	63.95	0.38	62.26	1.69	0.24	95.15	12.54	29.40	12.98	6.93

Table S2. DOM molecular indexes calculation formula

**Index**	**Formula**
Nominal Oxidation State of Carbon (NOSC)[9]	$\mathrm{NOSC} = \frac{4\mathrm{C} + \mathrm{H} - 3\mathrm{N} - 2\mathrm{O} + 5\mathrm{P} - 2\mathrm{S}}{\mathrm{C}} + 4$
Gibbs energies of the oxidation half reactions (ΔG^0_{Cox})[10]	$(\Delta G^0_{Cox}) = 60.3 - 28.5 \times \mathrm{NOSC}$
Double Bond Equivalent (DBE)[11]	$\mathrm{DBE} = 1 + 0.5 \times (2\mathrm{C} - \mathrm{H} + \mathrm{N} + \mathrm{P})$
Aromatic Index (modified) (AI_mod)[11]	$\mathrm{AI\_mod} = 1 + \mathrm{C} - 0.5\,\mathrm{O} - \mathrm{S} - \frac{0.5(\mathrm{H} + \mathrm{P} + \mathrm{N})}{\mathrm{C} - 0.5\,\mathrm{O} - \mathrm{S} - \mathrm{N\_P}}$

Table S3. Significantly enriched gene members of module 7 (one of the representative modules from the WGCNA analysis)

Gene ID	Description	GeneRatio	BgRatio	pvalue	p.adjust	qvalue	Count
ko05204	Chemical carcinogenesis - DNA adducts	0.05	0.004	1.44E-09	4.12E-07	3.79E-07	10
ko00830	Retinol metabolism	0.045	0.006	4.34E-06	0.0006233	0.0005738	9
ko00982	Drug metabolism - cytochrome P450	0.03	0.003	2.49E-05	0.0023853	0.0021959	6
ko04910	Insulin signaling pathway	0.05	0.011	8.07E-05	0.0057911	0.0053313	10
ko00591	Linoleic acid metabolism	0.025	0.003	0.0001174	0.0067391	0.0062039	5
ko05100	Bacterial invasion of epithelial cells	0.04	0.008	0.0001902	0.0090979	0.0083755	8
ko04917	Prolactin signaling pathway	0.035	0.007	0.0004536	0.0185992	0.0171223	7

References

1. Vollenweider, R. A., Giovanardi, F., Montanari, G. & Rinaldi, A. Characterization of the trophic conditions of marine coastal waters with special reference to the NW Adriatic Sea: proposal for a trophic scale, turbidity and generalized water quality index. *Environmetrics* **9**, 329–357 (1998).
2. Primpas, I. & Karydis, M. Scaling the trophic index (TRIX) in oligotrophic marine environments. *Environmental monitoring and assessment* **178**, 257–269 (2011).
3. Kang, J. *et al.* Biogeochemical factors regulating photosynthetically dissolved organic carbon produced by phytoplankton in the Taiwan Strait. *Environmental Research* **252**, 119090 (2024).
4. Utermöhl, H. Zur vervollkommnung der quantitativen phytoplankton-methodik: Mit 1 Tabelle und 15 abbildungen im Text und auf 1 Tafel. *Internationale Vereinigung für theoretische und angewandte Limnologie: Mitteilungen* **9**, 1–38 (1958).
5. Zhang, S. *et al.* Population dynamics and interactions of *Noctiluca scintillans* and *Mesodinium rubrum* during their successive blooms in a subtropical coastal water. *Science of The Total Environment* **755**, 142349 (2021).
6. Jin, D., Chen, J. & Huang, K. Marine Phytoplank Diatoms of China. Shanghai Scientific & Technical Publishers, Shanghai, China. 230p. (1965).
7. Yang, S. & Dong, S. Atlas of Common Planktonic Diatoms in China Sea Area. *Ocean University of China Press: Qingdao, China* 1–267 (2006).
8. Lu, D. *et al.* Causative species of harmful algal blooms in Chinese coastal waters. *Algological Studies* **145**, 145–168 (2014).
9. Riedel, T., Biester, H. & Dittmar, T. Molecular Fractionation of Dissolved Organic Matter with Metal Salts. *Environ. Sci. Technol.* **46**, 4419–4426 (2012).
10. LaRowe, D. E. & Van Cappellen, P. Degradation of natural organic matter: A thermodynamic analysis. *Geochimica et Cosmochimica Acta* **75**, 2030–2042 (2011).
11. Koch, B. P. & Dittmar, T. From mass to structure: An aromaticity index for high-resolution mass data of natural organic matter. *Rapid communications in mass spectrometry* **20**, 926–932 (2006).
